# Assessing pharmacy student experience with, knowledge of and attitudes towards harm reduction: illuminating barriers to pharmacist-led harm reduction

**DOI:** 10.1186/s12954-018-0262-6

**Published:** 2018-11-16

**Authors:** Lily Rowan Mahon, Amanda N. Hawthorne, Julie Lee, Heather Blue, Laura Palombi

**Affiliations:** Department of Pharmacy Practice and Pharmaceutical Sciences, University of Minnesota, College of Pharmacy, 232 Life Science, 1110 Kirby Drive, Duluth, MN 55812-3003 USA

**Keywords:** Naloxone, Harm reduction, Pharmacy, Education

## Abstract

**Background:**

As the burden from the opioid epidemic continues to increase in the state of Minnesota and across the nation, the University of Minnesota College of Pharmacy seeks to design an innovative, comprehensive harm reduction curriculum in order to better train student pharmacists to serve the varied needs of the greater community. This study examines incoming individuals’ baseline knowledge of and attitudes toward harm reduction in order to better inform curriculum planning and to ultimately produce pharmacists capable of impacting the devastating effects of the opioid crisis.

**Methods:**

Incoming first-year pharmacy students took a survey focused on their knowledge of opioid overdose and the drug naloxone and also provided written reflections on their perceptions of harm reduction. Data was coded using consensual qualitative research (CQR) into appropriate domains.

**Results:**

Pharmacy students beginning their professional education revealed a lack of knowledge of proper response to an overdose situation, with 18.56% unfamiliar with the opioid antagonist drug naloxone. Close to 10% (9.58%) of students expressed unwillingness to do anything other than call an ambulance during an overdose event, while 8.98% were either unsure or felt that they would not feel compelled to do something to help. Qualitative coding revealed many barriers to students’ becoming capable harm reductionists, including lack of knowledge of substance use, addiction, and harm reduction, in addition to the presence of bias and stigma.

**Conclusion:**

In order to interrupt the cycle of misinformation and stigma within the larger community and the subgroup of medical providers, gaps in student knowledge must be addressed in meaningful, specific ways over the course of their pharmacy education. Evaluating baseline knowledge and beliefs informs the design of a flexible, action-oriented curriculum to produce well-trained pharmacists ready to engage in finding solutions to the opioid crisis.

## Background

Long an issue of vital importance in the USA, opioid abuse and overdose has recently reached epidemic proportions, with 115 Americans dying every day from an opioid overdose [1]. Within Minnesota, opioids, including both prescription painkillers and illicit drugs such as heroin, have killed more than 2700 individuals in the last 15 years. Nearly 70% of these deaths involved prescription drugs, and more than half have occurred since 2013 [2]. There were 376 opioid-involved deaths in 2016, up 12% from 2015 [3].

The US Department of Health and Human Services (HHS) has declared its intention to address the opioid crisis through two overarching goals: firstly, to decrease opioid overdoses and associated mortality rates and, secondly, to decrease the prevalence of opioid abuse [4]. Pharmacists are ideally positioned to contribute toward these important public health goals as the interface between the community and the health care system.

The World Health Organization denotes pharmacists as the most easily accessible health professional [5]. Appointments are often not required to see a pharmacist, who can interact with dozens of patients daily. Furthermore, 93% of Americans live within 5 mi of a community pharmacy, many of which are open 12 to 16 h a day, 7 days a week [6]. The National Alliance of State Pharmacy Associations (NASPA) summarizes the expanding role of pharmacists: “There is growing recognition across the USA that pharmacists are a perfect resource to enhance access to public health services due to their expertise in medications and wellness and their exceptional accessibility [7].” This accessibility can be leveraged to greatly benefit many rural and underserved populations impacted by the opioid crisis, particularly in the increasingly vital realm of harm reduction.

Although controversial in some countries [8, 9], harm reduction methods have proven internationally effective in their goal of mitigating certain health risks. Despite the fact that access to naloxone in the USA has been associated with a 9 to 11% decrease in overdose deaths [10], the USA arguably lags behind other countries where harm reduction has been well-established for many decades. The first supervised injection facility (SIF) in North America, located in Vancouver, British Columbia, has been estimated to save the public over 13 million American dollars annually in costs associated with HIV diagnoses and deaths [11], all while providing critical support services to people who inject drugs and decreasing public exposure to injection paraphernalia [12]. A hypothetical safe-injection facility in a major US city has been calculated to save upwards of 5 million dollars while bringing about 121 people into treatment [13]. As for syringe exchange programs, an extensive review has found that such programs are documented as among the most impactful and cost-effective of harm reduction initiatives [14]. As the USA, struggles with finding solutions to the opioid crisis, which continues to claim lives in record proportions and has cost the country over one trillion dollars [15], harm reduction must be pursued as a solution regardless of controversy. As highly trained and accessible health professionals, pharmacists must equip themselves with knowledge of harm reduction tools in order to promote healthy communities. Thanks to new legislation in several states opening access to harm reduction initiatives, particularly naloxone availability, it is a simpler and more necessary task than ever.

In Minnesota, the Opiate Antagonist Protocol was passed during the 2016 Regular Session, granting pharmacists and other medical providers greater capability to dispense naloxone [16]. Together with the Syringe Access Initiative of 1998 and authorized collector of unwanted pharmaceuticals legislation of 2016, Minnesota pharmacists have a wider breadth of harm reduction tools. Despite the fact that Minnesota has legislation that supports pharmacists to increase access to naloxone and clean syringes, these harm reduction approaches have unfortunately not been widely adopted in Minnesota (research by Palombi L et al., unpublished data, 2018).

The University of Minnesota College of Pharmacy, recognizing the robust role pharmacists could and should play in engaging with harm reduction to reduce morbidity and mortality resulting from the opioid crisis [17, 18] and the impact that pharmacy curricula advancements can make in pharmacy practice, made harm reduction education a greater priority in the PharmD curriculum. Starting in the autumn of 2017, pharmacy students were introduced to the opioid crisis, the drug naloxone and its role in reversing opioid overdose, as well as other harm reduction approaches in their first semester of study. Substance use education was subsequently expanded stepwise in consecutive years of the curriculum.

In response to the devastating toll of the opioid epidemic and such expanded legislation, 94 pharmacy schools within the USA have publicly committed to educating each student pharmacist on prevention and treatment of prescription drugs of abuse [19]. Per the American Association of Colleges of Pharmacy (AACP), each student will be educated on life-saving overdose interventions, including the opioid antagonist naloxone, and how to counsel patients, individuals, and the general public on appropriate use of these medications. The University of Minnesota is one of the 94 colleges that has taken this pledge [19]. This increased focus on the opioid epidemic should enhance the professional career of pharmacists—and their role within public health. Pharmacists can utilize Collaborative Practice Agreements (CPAs), point-of-care (POC) testing, patient consultations, monitoring the prescription drug monitoring program, and be a point of contact for patients to find resources from specialty care to safe disposal of medications [20]. While acknowledging the diverse role that pharmacists can play, this paper’s main focus will be on the most common harm reduction measures in pharmacy practice, needle exchange and the opioid antagonist drug naloxone.

In the USA, pharmacy education entails a doctoral program that requires extensive prerequisites in the fields of health care and the sciences. Most students enter the program with a Bachelor’s degree, thus having a higher level of education than the public average with baseline knowledge in health-related subjects, yet usually lacking the real-world practicing experience of a medical professional. Incoming pharmacy students, like other pre-professional medical students, therefore stand at an intriguing point in their careers worthy of exploration. They enter their professional program with many beliefs and perceptions swayed by public opinion, with the intention of graduating their program as knowledgeable, informed practitioners, a goal the university holds, as well. It is thus in the interest of both universities and the pharmacy profession to gauge baseline attitudes and beliefs of its incoming students concerning opioid addiction and harm reduction so that curriculum can be tailored to address their gaps in knowledge, build upon their strengths, and create better trained pharmacists adept at further educating their patients on this important public health need.

Although previous literature has examined the attitudes of medical students and practicing physicians toward harm reduction [18, 21–25] and the effectiveness of student learning initiatives focused on harm reduction, there is a gap in the literature pertaining to perceptions individuals hold as they enter professional programs, particularly pharmacy, the underutilized health professional [26]. Survey tools, including the Opioid Overdose Knowledge Scale (OOKS) and the Opioid Overdose Attitudes Scale (OOAS), were created and internally validated to evaluate take home naloxone training in a standardized format for healthcare professionals and for individuals who are using opioids [27]. Additionally, the Brief Opioid Overdose Knowledge (BOOK) survey is a 3-factor 12-question survey that is an abbreviated form of the OOKS that has been validated with participants who were both illicit and licit opioid users [28]. Use of these questionnaires allows for a standardized way to assess student knowledge. To date, no studies have combined incoming student knowledge with a qualitative analysis of their attitudes and perceptions of harm reduction. Such information provides a useful baseline to inform curriculum and efforts to steer pharmacy practice, as well as to diagnose the impact of public attitudes on the field of pharmacy. In order to better educate and engage student pharmacists, this study explored University of Minnesota College of Pharmacy first-year pharmacy students’ preparedness to respond to an overdose situation and their knowledge and attitudes toward harm reduction methods for prescription and illicit opioid use, particularly naloxone distribution and syringe exchange.

## Methods

Quantitative data focused on incoming pharmacy student knowledge of opioid overdose and student confidence in using naloxone. Data was collected prior to a 1-h presentation on the pharmacist’s role in public health and an introduction to harm reduction. Following the presentation, qualitative data was collected from student pharmacists’ written reflections responding to prompts concerning beliefs on addiction and harm reduction. The sequence of data collection ensured that quantitative data provided baseline knowledge of incoming student pharmacists’ knowledge and attitudes toward opioid misuse, thus informing educational efforts tailored to bridge knowledge gaps. Qualitative data was collected after the presentation so that students would have knowledge of the fundamentals of harm reduction thus be able to provide educated reflections.

### Quantitative survey

A survey instrument was developed by adaptation of the OOKS, OOAS, and the BOOK questionnaire. It was then administered over an anonymous online Qualtrics platform. The surveys reached first-year pharmacy students at both the Minneapolis and Duluth campuses of the University of Minnesota College of Pharmacy, totaling 167 responses. The survey was conducted in the first month of pharmacy school, before students were taught about the pharmacists’ role within public health and given an introduction to harm reduction. A 99.4% response rate was achieved.

Participants completed the BOOK questionnaire, consisting of 12 questions rated on an ordinal scale (available responses were “True,” “False,” and “I Don’t Know”) to discourage random guessing and reduce the chance that participants may accidentally answer an item correctly. In addition, participants completed an extensive 29-item OOAS that was anchored by a five-point Likert-type scale (1 = completely disagree, 2 = disagree, 3 = unsure, 4 = agree, and 5 = completely agree) with the most relevant of the 29 OOAS presented in the results. For all quantitative measures, the five-point Likert scale was reduced to a three-point scale by consolidating the “completely agree” and “agree” responses into one “agree” response and the “completely disagree” and “disagree” responses into “disagree.”

### Qualitative data collection

Student pharmacists submitted written reflections after listening to a lecture focused on the public health impact of the opioid crisis (1 h) and engaging in a brief discussion on harm reduction and the pharmacist’s role within public health (1 h). Written reflections allowed student pharmacists to address how the lectures and discussions within their first month of pharmacy curriculum informed their understanding of substance use disorder, contributed to their professional and personal development, and inspired them to promote public health and harm reduction initiatives. Students were asked to consider the following three questions in a reflection:

1. Describe how you feel, in general, about harm reduction approaches to substance use.
2. What is your personal opinion on dispensing needles? Is this different or the same than your professional opinion? Why or why not?
3. What is your personal opinion on dispensing naloxone? Is this different or the same than your professional opinion? Why or why not?

Reflection questions were formulated by a small group of faculty who taught harm reduction and were familiar with conflicting student and pharmacist views on harm reduction. The reflection assignment was not restricted to a specific page length, and student pharmacists were encouraged to share personal perspectives in a way that was comfortable to them. Students were made aware that their grade was not based on their stance, only on their completion of the paper.

The assignment was given to all first-year pharmacy students at both the Minneapolis and Duluth campuses of the University of Minnesota College of Pharmacy; a 100% response rate was achieved. Before qualitative analysis began, all papers were de-identified by one of the coders, replacing students’ names and campuses with a student number (SN). The University of Minnesota IRB determined that this study was not human research.

### Qualitative analysis

Using the principles of Consensual Qualitative Research (CQR), two coders reached consensus among domains and categories based on the research questions and study aims. Coders were PharmD candidates in their second and third years. Two pharmacist-faculty preceptors served as auditors and provided feedback throughout the process. Table 1 summarizes the CQR process.

**Table 1:** The consensual qualitative research process

Coding cycle	Coding step	Description of coding step
First cycle	CQR: Independent holistic coding	• Transcripts were de-identified, including students name and campus. • Read transcriptions • Identify topic areas that represent what the participants say • Circle or section off blocks of the narrative that fall under a particular topic area • Identify or propose a domain name for that topic area
	CQR: Meeting #1	• Discuss how each team member reached domain names by discussing raw data • Reach consensus on domain names • Reach consensus on core ideas • Discuss how each team member decided on category names by discussing data presented in table • Reach consensus on categories
	Code mapping (first iteration)	• Compile all domains with the corresponding blocks of data • Develop core ideas to include in table format • Sort the table by domain in order to have all like domains next to one another • Auditor reviews list to finalize first iteration table
Second cycle	CQR: Independent descriptive coding	• Read transcripts for a second time • Color code sections that were placed in domains so that each coder could confirm they agreed with the placement.
	Model generation	• Organize domains and categories using a Model of Critical Pedagogy to create a visualization of important themes and information • Final organization of domains and subdomains to allow for best sense presentation

The CQR process started with holistic coding in the first in-person round to identify themes in sections of text/paragraph in larger sections [29]. To detect themes that related to the impact pharmacists can have in harm reduction, coders looked for reflections that addressed the student pharmacists’ experiences and emotions as a result of the lecture relating to public health, biases, or perceptions that the experience uncovered and/or challenges, and changes in student pharmacist thinking. Initial domain themes were independently identified for segments of raw data by each coder. Larger segments of data were coded as a whole instead of coding line by line to address the study’s research questions.

Domains were cross-analyzed and used for the next step in code mapping [29, 30]. The code list was further updated after consultation with the auditors. Descriptions of domain themes and coding subdivisions were identified to better accommodate the different writing styles of students (Table 2). The auditors reviewed and approved the domains and descriptions prior to the second round of CQR.

**Table 2:** Domain themes, descriptions, and coding subdivisions after second cycle coding

Domain	Domain themes and description	Coding subdivisions
Domain I	Education: Student describes importance of education in harm reduction for student, patients/community, and pharmacist.	• Curriculum/Student view • Patients/community • Pharmacist
Domain II	Dispensing Needles: Student describes their opinions on dispensing needles without prescription	• Personally opposed but professionally for • Professionally opposed but personally for • Personally and professionally for • Personally and professionally against
Domain III	Dispensing naloxone: Students describe their opinions on dispensing naloxone	• Personally opposed but professionally for • Professionally opposed but personally for • Personally and professionally for • Personally and professionally against
Domain IV	Past working experience in the pharmacy: Students describe their past working experiences in the pharmacy that relate to needle dispensing, naloxone dispensing, or Prescription Monitoring Program (PMP)	• No subdivisions in this domain
Domain V	The role of the pharmacist: Student describes pharmacists’ role in harm reduction approaches to substance use	• Open minded • Non-judgmental • Recognizing stigma addiction/mental health • Communication/interprofessional • Public health role
Domain VI	Barriers within students: Student describes the information related to dispensing naloxone in an incorrect manner and/or misunderstands the questions	• Would be okay with giving out for prescription but not for illicit drugs • Incorrect knowledge • Misunderstand the questions • Miscellaneous viewpoints
Domain VII	Past experiences and exposures: Student describes their past experiences that relate to addiction and people who have/are addicted	• No subdivision in this domain
Domain VIII	Leading cause of opioid epidemic: Student describes their beliefs on major leading cause of opioid epidemic in the USA	• Overprescribing • Manufacturing the opioid • Lack of education toward opioids • Prescription drugs increased use of illegal street drugs
Domain IX	Solutions: Student describes the possible solution to the opioid epidemic	• No subdivision in this domain

After consensus was reached on domains and their descriptions, the two coders worked together and completed a second final round of coding. Themes became more descriptive in the second round of coding, and codes were identified in a line-by-line fashion. This descriptive coding process allowed for organization of domains around the study aims [29]. After the coding process and during the final write up, subdomains were resorted to allow for the most consistent presentation of the data.

## Results

The abbreviated BOOK questionnaire data is illustrated in Table 3. BOOK *factor 1*, general opioid knowledge, showed that incoming first-year pharmacy students showed limited and incomplete knowledge of opioid overdose. When asked if long-acting opioids could treat long-term pain, 17.96% of students answered incorrectly. In regards to *factor 2*, which addressed opioid overdose risk knowledge, 16.76% of students believed—incorrectly—that all overdoses are fatal. When asked if trouble breathing is a symptom of opioid overdose, 17.97% responded that it was not or that they did not know. Overall, students had a lower percentage accuracy rate for *factor 2* than *factor 1*, along with an increased reporting of uncertainty. *Factor 3* focused on opioid overdose response knowledge. Less than half of respondents (47.31%) reported being aware of the role of rescue breathing, and only 31.13% correctly identified a sternal run as an easy and effective means of determining if one is unconscious, a cardinal sign of overdose. Additionally, 18.56% reported that they were not familiar with naloxone, the principal pharmaceutical response to the opioid crisis.

**Table 3:** Student reported brief opioid overdose risk knowledge

	Correct (%)	Incorrect (%)	I do not know (%)
Factor 1: General opioid knowledge			
Long-acting opioids are used to treat chronic “round the clock” pain. [Statement is true]	124 (74.25)	13 (7.78)	30 (17.96)
Methadone is a long acting opioid. [Statement is true]	48 (28.74)	32 (19.16)	87 (52.10)
Restlessness, muscle and bone pain, and insomnia are symptoms of opioid withdrawal. [Statement is true]	129 (77.25)	5 (2.99)	33 (19.76)
Heroin, OxyContin(R), and fentanyl are all examples of opioids. [Statement is true]	141 (84.43)	4 (2.40)	22 (13.17)
Factor 2: Opioid overdose risk knowledge			
Trouble breathing is NOT related to opioid overdose. [Statement is false]	137 (82.04)	2 (1.20)	28 (16.77)
Clammy and cool skin is NOT a sign of an opioid overdose. [Statement is false]	107 (64.07)	5 (2.99)	55 (32.93)
All overdoses are fatal (deadly). [Statement is false]	133 (79.64)	28 (16.77)	6 (3.59)
Using a short-acting opioid and a long-acting opioid at the same time does NOT increase your risk of an opioid overdose. [Statement is false]	127 (76.04)	5 (2.99)	35 (20.96)
Factor 3: Opioid overdose response knowledge			
If you see a person overdosing on opioids, you can begin rescue breathing until a health worker arrives. [Statement is true]	74 (44.31)	14 (8.38)	79 (47.31)
A sternal rub helps you evaluate whether someone is unconscious. [Statement is true]	52 (31.14)	10 (5.99)	105 (62.87)
Once you confirm an individual is breathing, you can place him/her in the recovery position. [Statement is true]	100 (59.88)	12 (7.19)	55 (32.93)
Narcan (naloxone) will reverse the effect of an opioid overdose. [Statement is true]	131 (78.44)	5 (2.99)	31 (18.56)

The OOAS survey (Table 4) gauged students’ attitudes and concerns toward naloxone use, in which 92.22% of the students reported that they would need further training before feeling comfortable handling an overdose situation. Sixty-four percent of students expressed that they would feel safer if naloxone was around. Most students expressed comfort with calling emergency services (92.81% unconcerned with calling emergency personnel for fear of police presence) and applying chest compressions (84.43%). Twenty-five percent (25.19%) would be afraid to use naloxone for fear of the recipient becoming aggressive afterward, and another 17.36% for precipitating withdrawal symptoms. Overall, only 12.57% agreed that if someone overdosed, they would know what to do to help them. Close to 10% (9.58%) of students expressed unwillingness to do anything other than call an ambulance during an overdose event. Similarly, 8.98% again were either unsure or felt that they would not feel compelled to do something to help. More pertinent to pharmacy practice rather than a crisis situation, 36.52% agreed that everyone at risk of witnessing an overdose should be given naloxone, while 27.55% disagreed, and 35.93% were unsure.

**Table 4:** Student reported opioid overdose attitudes scales responses—abbreviated

	Agree (%)	Disagree (%)	Unsure (%)
I am already able to inject naloxone into someone who has overdosed.	6 (3.59)	154 (92.22)	7 (4.19)
I am going to need more training before I would feel confident to help someone who has overdosed.	154 (92.22)	6 (3.59)	7 (4.19)
If someone overdoses, I would know what to do to help them.	21 (12.58)	90 (52.89)	56 (33.53)
I know very little about how to help someone who has overdosed.	111 (66.47)	30 (17.96)	26 (15.57)
I would be afraid of giving naloxone in case the person becomes aggressive afterword.	42 (25.15)	68 (40.72)	57 (34.13)
I would be afraid of doing something wrong in an overdoses situation.	130 (77.84)	18 (10.78)	19 (11.38)
I would be reluctant to use naloxone for fear of precipitating withdrawal symptoms.	29 (17.37)	95 (56.89)	43 (25.75)
I would feel safer if I knew that naloxone was around.	107 (64.07)	18 (10.78)	42 (24.15)
I would be afraid of suffering a needle stick injury if I had to give someone a naloxone injection.	42 (25.15)	93 (55.69)	32 (19.16)
Needles frighten me and I wouldn’t be able to give someone an injection of naloxone.	14 (8.38)	141 (84.43)	12 (7.19)
Everyone at risk of witnessing an overdose should be given a naloxone supply.	61 (36.53)	46 (27.55)	60 (35.93)
I couldn’t just watch someone overdose, I would have to do something to help.	147 (89.02)	4 (2.40)	16 (9.58)
If someone overdoses, I would call an ambulance but I wouldn’t be willing to do anything else.	16 (9.58)	118 (70.66)	33 (19.76)
Family and friends of drug users should be prepared to deal with an overdose.	149 (89.02)	8 (4.79)	10 (5.99)
If I saw an overdose, I would panic and not be able to help.	11 (6.59)	122 (73.05)	34 (20.36)
If I saw an overdose, I would feel nervous, but I would still take the necessary actions.	145 (86.83)	7 (4.19)	15 (8.98)
I will do whatever is necessary to save someone’s life in an overdose situation.	148 (88.62)	4 (2.40)	15 (8.98)
If someone overdoses, I want to be able to help them.	158 (94.61)	2 (1.20)	7 (4.19)
I would be concerned about calling emergency services in case the police come around.	5 (2.99)	155 (92.81)	7 (4.19)
I would be able to perform chest compressions to someone who has overdosed.	141 (84.43)	10 (5.99)	16 (9.58)

The CQR analysis (Table 1) of qualitative data resulted in ten domain themes, illustrated in Table 2. Table 5 highlights final coding results by domain/subdomain, mention, and frequency. Table 6 highlights examples of reflection discussions by domain.

**Table 5:** Final coding results by domain/subdomain, mention, and frequency

Domain	Sub domain	Number of times mentioned	Subdomain Percentage of total mentions	Domain Percentage of total mentions
Domain I Education	Curriculum/student view	61	7.4%	20.0%
	Patients/community	88	10.6%	
	Pharmacist	17	2.1%	
Domain II Dispensing Needles	Personally opposed but professionally support	29	3.5%	19.5%
	Professionally opposed but personally support	6	0.7%	
	Personally and professionally for support	121	14.6%	
	Personally and professionally opposed	6	0.7%	
Domain III Dispensing Naloxone	Personally opposed but professionally support	8	1.0%	19.6%
	Professionally opposed but personally support	2	0.2%	
	Personally and professionally support	151	18.2%	
	Personally and professionally opposed	2	0.2%	
Domain IV Past work experience	No subdomains	34	4.1%	4.1%
Domain V The role of the pharmacist	Open minded	6	0.7%	25.9%
	Non-judgmental	46	5.5%	
	Recognizing stigma addiction/mental health	65	7.8%	
	Communication/interprofessional	19	2.3%	
	Public health role	79	9.5%	
Domain VI Barriers within students	Would be okay with giving out for prescription but not for illicit drugs	3	0.4%	2.4%
	Incorrect knowledge	8	1.0%	
	Misunderstand the questions	1	0.1%	
	Miscellaneous views	8	1.0%	
Domain VII Past experiences and exposures:	No subdomains	22	2.6%	2.6%
Domain VIII Leading causes of opioid epidemic	Overprescribing	11	1.3%	3.9%
	Manufacturing the opioid	4	0.5%	
	Lack of education toward opioids	5	0.6%	
	Prescription drugs → increased use of illegal street drugs	13	1.6%	
Domain IX Potential solutions	No subdomains	14	1.7%	1.6%

**Table 6:** Example of reflection discussions by domain

Domain	Sub domain	Quotes (SN = student number)
Domain I Education	Curriculum/student view	“I am a perfect example of how education can help because I had the preconceived notions mentioned above on how these programs can be harmful before I heard the presentation in class and before I read the articles on them.” SN 156
	Patients/community	“I think education is crucial if we want to reduce substance abuse.” SN 113
	Pharmacist	“If I had more training on how to help someone in an overdose situation besides calling 911 and knowing that I should [use] naloxone, I would feel more comfortable dispensing naloxone to my patients because then I could counsel them on how to use it as well.” SN 24
Domain II Dispensing Needles	Personally opposed but professionally support	“I believe that syringes should be used for medical purposes only and that they should be reserved for those who need to use them in the treatment of a medical condition. My younger brother is a Type 1 diabetic and has a need to use syringes for the maintenance of his diabetes. So, if a pharmacy dispenses needles to people without prescriptions that would mean less inventory for those who need needles to treat an actual medical condition such as insulin dependent diabetes, like my younger brother.” SN 53 “On a personal level, I feel that by dispensing more needles, more used needles will accumulate on those playgrounds, making the likelihood of children developing HIV/AIDS much higher.” SN 126
	Professionally opposed but personally support	“I have to follow the rules as well as the practice that I have learned. Freely dispensing needles can cause problems for the society since we cannot control how many needles and syringes are in used.” SN 161
	Personally and professionally for support	“By default, I think that pharmacies should dispense needles and that they should do so until it becomes a public health concern for the overall population. In this case, I think that my personal and professional stance are very similar. I personally am a little conflicted in saying that all pharmacies should dispense needles to anyone that asks for them, because there are potentially harmful consequences of that stance. While at the same time with the right programs put into place, sharps containers being available in public places and promoting treatment, the outcome of a reduction in infectious diseases would be a benefit to overall public health.” SN 134
	Personally and professionally opposed	“Although, there has been studies shown that access to needle reduces transmission of infections I think otherwise.” SN 136
Domain III Dispensing Naloxone	Personally opposed but professionally support	“When you have a close friend who passed away from having an adverse reaction from being served a meal he was allergic to and did not have the chance to use an epinephrine pen to save his life, you begin to wonder why should someone abusing drugs be given a ‘just-in-case’ card to save their life.” SN 51
	Professionally opposed but personally support	“I feel the cost of treating and preventing an overdose should fall on the patient because they made a life choice to take a drug inappropriately and against doctors’ orders. I do not feel like naloxone should not be accessible though.” SN 21
	Personally and professionally support	“My personal opinion is that naloxone should be dispensed freely through pharmacies, because in order to treat drug-reliant individuals, we first need to keep them alive.” SN 31 “I do not think it encourages risky behavior, rather, it provides patients with a backup to use in emergencies. For instance, I do not think dispensing an epi pen encourages patients to encounter their allergens, but it serves as a vital potential lifesaver in case of an emergency.” SN 91
	Personally and professionally opposed	“I believe we cannot be making excuses to a drug problem by counteracting it with another drug that might in the end promote the abuse more. Naloxone will only promote overall opioid substance use.” SN 136
Domain IV Past work experience	No subdomains	“I have started to implement this already at my current pharmacy intern position by creating syringe kits to sell to patients that request them. In these kits, patients get 10 syringes, a sheet on safe needle disposal, information on how to acquire and administer naloxone, a list of phone numbers for treatment centers, and the national suicide hotline. Having this information in the kits is a discrete, non-judgmental, and pressure free way to get patients the information that could help them on the road to recovery.” SN 149 “I have a certain bias that comes from the pharmacy [...]We did not dispense needles without an injectable prescription on file, and I believe that it was the correct thing to do for that location. The pharmacy is located directly on a green line station, meaning we had people from all over the twin cities coming to our store. If that store dispensed needles, the amount of problems it would create would not outweigh the benefits of potentially reducing disease transmission or infection. However, I am completely onboard with other stores dispensing needles, if they are distributing them to a population base that truly needs it.” SN 159 “Many of the people I sell syringes to already appear dangerously high, and a few weeks ago we had to close the pharmacy and call an ambulance as a syringe customer overdosed and began seizing in our parking lot. Another day, though, when I asked the pharmacist if we should hold a patient’s hydrocodone prescription because she was slurring her words and had white powder around her nose, the pharmacist told me that we did not have the right to determine whether or not she needed her prescription and that it would be an infringement on her privacy to make any assumptions.” SN 110
Domain V The role of the pharmacist	Open minded	“I will have an open mind as to why people need clean needles and to take the time to talk to them without judgment so they feel that they can trust me enough to come for help when needed.” SN 84
	Non-judgmental	“They are still people, their addiction does not define them or who they are as person. It would be unethical and cruel to ignore the medical risks my patients who struggle with addiction face because it would mean giving them a lesser quality of care simply because of their addiction status” SN 149
	Recognizing stigma addiction/mental health	“People are all people, whether they have an addiction or not. We all crave compassion and for someone to listen.” SN 19
	Communication/interprofessional	“Healthcare professionals and government officials need to learn how to communicate with one another because both sides depends on the other when it comes to keeping our communities healthy” SN 19
	Public health role	“I believe that all pharmacists should have the public health mindset that overall population livelihood comes before personal beliefs. Naloxone can prevent death, and I do not believe one gets to ‘play God’ by deciding someone who uses (or anyone for that matter) does not deserve to be saved.” SN 33
Domain VI Barriers within students	Would be okay with giving out for prescription but not for illicit drugs	“I believe if a patient needs needles they should have to show evidence of either a past prescription that indicates they are still using the needles or some documentation that provides that the patient requires the needles for a medical reason. If a patient cannot show documentation of why they need the needles then they most likely do not need the needles and will be using them to shoot heroin.” SN 30
	Incorrect knowledge	“As for the naloxone dispensing, I do not support the idea that it should be wildly available to citizens. Naloxone is an opiate antagonist which is used intravenously in emergency situations to reverse the respiratory depression caused by overdoses of heroin, morphine or other opioids.” SN 165
	Misunderstand the questions	I am only half agree with the statement of the former FDA head that “Opioid epidemic is one of ‘the great mistakes of modern medicine.’ The reason for my half agreement is that at the time opioid was invented, none would think that humans would addict to it. The medication was purely intended for relieving pain, but humans figured another way to abuse the substance and eventually die from overdose.” SN 67
	Miscellaneous views	“Ultimately I believe that opioid and other narcotic abusers can get well, but they need to have a strong enough desire to quit, and unfortunately that generally does not happen until the impact of their drug abuse has caused massive consequences that force them to examine their choices. As a result, on a personal level, I feel that harm reduction approaches to addiction, like needle exchange, can actually do more harm than good.” AN 103
Domain VII Past experiences and exposures to addiction	No subdomains	“I recently lost a family member to a prescription drug overdose, but there was no antidote for that drug. My family was still devastated by our loss even though we knew the doctors/pharmacists did everything they could to save this family member. I cannot even imagine how devastating it would be for someone else to lose a family member to an opioid, knowing that an antidote does exist yet they did not have it accessible at the right time.” SN 18 “Personally, my good friend has been saved twice because naloxone was easily available to be purchased and was kept in her home. If naloxone was not available to be purchased by the general public, I am confident that my friend would no longer be alive.” SN 102 “It’s also important for me personally because as someone who struggles with mental health disorders, though they are not related to addiction, people treat you differently. They see you as broken and pity you or even get angry because they do not understand why you do the things you do.” SN 149
Domain VIII Leading causes of opioid epidemic	Overprescribing	“Overprescribing of opioids is what sets the path for addiction in many patients.” SN 1
	Manufacturing the opioid	“What we are hoping to see is the actions taken in reducing the manufacturing the opioids because those companies are facing decreases in profit if the consumption of opioids goes down.” SN 11
	Lack of education towards opioids	“My doctors did not even ask me about the pain, that they did not try to stop me from taking those powerful drugs, and I did not realize that I was getting addicted until three weeks later.” SN 82
	Prescription drugs → increased use of illegal street drugs	“As it becomes harder to obtain prescription opioid both legally and illegally, more individuals are moving to heroin which is cheaper and more addictive.” SN 49
Domain IX Potential solutions	No subdomains	“I feel that when a patient is found abusing substances and instead of taking that person to jail, they should be taken to a rehab facility instead” SN25 “I believe in my future pharmacy practice these vending machines could be placed in the pharmacy and I could provide patient education on safety of needles, risks of drug use, and options for treatment programs” SN 21

Domains 1, 2, and 3, which focused on education, dispensing naloxone, and dispensing needles, were well represented in the data. These three domains included the three most frequently cited subdomains, with 18.2% of all citations representing students who both personal and professional support for dispensing naloxone, 14.6% who personally and professionally support for dispensing needles, and 10.6% emphasizing the need for education for patients and the wider community. Domain 5, which focused on the role of the pharmacist, was the most frequently discussed domain representing 25.9% of all the responses that were coded. As for students’ perceptions of the role recognizing traits or beliefs necessary to be an effective harm-reduction pharmacist, including identifying stigma toward addiction (65) and taking a non-judgmental approach (46).

A number of students (22) reported on how they personally were affected by addiction; this was captured in domain 7. In addition, 34 student pharmacists provided relevant experiences from past work in pharmacy or related fields. Domain 6 captured student reflections that hinted toward negative attitudes or bias. Table 6 lists student quotes that stood out to the coders and/or well represented the domains/subdomains.

## Discussion

Opioids and substance use have become a recent focus of adaptive medical [21, 31], nursing [32–34], and pharmacy [35–37] curricula. This study utilized measures by which to assess incoming student knowledge and attitudes with regard to opioids and substance use. In doing so, it illuminated areas of further work in engaging student pharmacists in harm reduction throughout their education, while also affording a glimpse into contemporary pharmacy practice. Qualitative and quantitative data buttressed one another in order to inform a comprehensive addiction education curriculum and to gain potential insight to the current field of practice in pharmacy.

The BOOK and OOAKS surveys revealed that student pharmacists exhibited considerable knowledge gaps in addiction basics, overdose risk factors, and opioid overdose first-aid procedures. This is concerning, as students starting their first semester of the PharmD program at the University of Minnesota should have demonstrated basic competency in understanding the pharmacists’ role in public health and should have a basic understanding of the pharmacists’ public health role in the opioid crisis. This is also concerning in that the opioid crisis has gained a great deal of press and should have the attention of all pre-health professions students; approximately 46 people a day died from prescription opioid overdoses in 2016 [38] and heroin overdose deaths increased fivefold from 2010 to 2016 [39]. Due to these alarming numbers, the Surgeon General has issued an advisory that everyone at risk of witnessing an overdose, including community members and health care providers, carry naloxone [40]. With the access that pharmacists have to the public, it is vital that they have the knowledge of overdose risk factors and the first aid response for an opioid overdose. Any pharmacy curriculum seeking to educate student pharmacists on critical public health issues would deem these knowledge gaps a critical place to start an opioid-focused curriculum thread. BOOK and OOAS results revealed that the majority of students felt unprepared to handle an overdose situation. This is not surprising given that the survey was delivered before formal lectures had been received on the topic. There was consistently poor understanding of how to appropriately handle an overdose situation; even though these are healthcare professional students, they were not aware of basic first aid measures or of the role of naloxone. While these students have not yet taken pharmacy level graduate courses, they would have taken the mandated cardiopulmonary resuscitation (CPR) course, and additionally have been preparing to be health care professional students for at least 2 to 4 years. The fact this highly motivated population is not familiar with naloxone indicates a gap within the general public that sorely needs to be educated. Additionally, quantitative data revealed some presence of stigma toward those who misuse substances.

Notable aspects of survey results came from the biases that were discovered in coding student reflections. Not all students within this professional school felt compelled to help individuals who were suffering from addiction and from opioid dependency. The qualitative data was able to add an insightful addition to that finding as many personal stories were relayed in the reflections, giving reasonings behind these individuals’ decisions and opinions. There were some cultural and religious reasons which were used to explain some students’ views toward harm reduction, while others had personal tragedy with family and friends. Other students admitted in their papers that their beliefs had changed after lecture succinctly summarized as (SN 156), “I am a perfect example of how education can help because I had the preconceived notions mentioned above on how these programs can be harmful before I heard the presentation in class and before I read the articles on them.” Another student (SN 168) noted that, “My biggest takeaway from the lecture and this self-reflection is not just changing my view on such practices as dispensing needles and naloxone, rather as a health professional to provide a welcoming and nonjudgmental atmosphere for all of my patients thereby promoting an open and honest dialogue between them and myself.”

As for qualitative data, domains 1, 2, and 3 (which were focused on education, naloxone dispensing, and the dispensing of needles) were represented in nearly every student reflection, most likely due to the prompts that the students were asked to address. Domain 5, which focused on the role of the pharmacist, was the most frequently mentioned domain and is of particular interest because while students were given direction to discuss harm reduction measures, they also were asked to share their own opinions.

Student pharmacist reflections suggested that this group saw prescribing practices as the leading causes of the epidemic, with 13 mentions from student pharmacists on the cause and effect relationship for patients who take prescription painkillers turning to illicit drugs. As one student (SN 3) stated:“There is clearly a positive correlation between the amount of prescribed opioids and the number of opioid related overdose deaths.”

There were 11 students who directly addressed the problems associated with addiction evolving from providers overprescribing. One student (SN 60) articulated this thought as follows: “A lot of people become addicted to drugs from first starting out with a prescription given to them by their physician. These patients may not understand the seriousness of the drug, and may not even be aware that they are slowly becoming addicted. Once they run out of their prescription and it becomes harder to get a new one, many will turn to heroin.” Five students identified a lack of general education/awareness of opioids could have led to the epidemic that the USA is currently experiencing. This is especially interesting because in domain 1, there were 88 students who discussed the importance of pharmacists role within educating the community on harm reduction measures. One student (SN 82) shared a personal experience about an injury they had acquired and, distressingly, how inappropriately their physician handled prescribing opioids, “My doctors did not even ask me about the pain, that they did not try to stop me from taking those powerful drugs, and I did not realize that I was getting addicted until three weeks later.”

Students who reported being unsupportive of harm reduction reported having reasons similar to those observed in the pharmacist population, including beliefs that harm reduction promotes substance use [18]. Examples of direct statements include: “In my personal opinion, I don’t believe that pharmacies should dispense needles without a prescription. I believe this to be enabling a serious problem that is plaguing the United States. I would rather not help somebody who is looking to further harm themselves with the use of illicit drugs, such as heroin” (SN 53) and “I disagree with the fact that naloxone can be used as a kind of crutch for drug users. If they know they can be saved from an overdose by naloxone, then they may overdose more often” (SN 51).

Ideally, at the conclusion of their pharmacy education, the student pharmacists surveyed will be able to counsel clients effectively and sensitively on naloxone and other harm reduction methods and answer any questions and concerns they have. According to the above data, attaining such a knowledge base would require bridging significant gaps in knowledge of naloxone’s biological effects, legal and policy implications, stigma and/or bias, and the proven effectiveness of harm reduction. Interestingly, students who wrote these reflections had already been instructed on all of these topics with the exception of stigma.

Student pharmacists expressed fairly widespread support, both personally and professionally, for dispensing naloxone. Interestingly, support for dispensing needles was, although still in the majority, more tepid. This could stem from the separate phases in which naloxone and needles are used. Naloxone saves lives through its reversal of overdose effects *after* a drug has taken effect, and furthermore, its use can extend to those who are not using illicitly—for example, elderly patients taking prescription painkillers. On the other hand, needles are a widely known implement for drug injection. As many correlate syringe exchanges with enablement, lack of public support has historically undermined efforts to install exchanges nationwide [41]. However, some students do view naloxone in a similar light of perpetuating misuse, as will be discussed later.

Student pharmacists identified the dissemination of information about naloxone and substance abuse to patients and the wider community as critical to mitigating the epidemic (10.6% of total codes). Despite this and students’ widely held view of pharmacists as public health experts (9.6% of total codes), a belief also evident within practicing pharmacists [18], students interestingly did not frequently identify community engagement or education as a significant component of the public health role (2.3% of total codes). Schools of pharmacy are increasingly mindful of the professional and public health impacts of community engagement and inclusive of community-oriented opportunities in curriculum. This is essential for molding student pharmacists’ view of the pharmacist as more than a medication dispensary, but a holistic promoter of health.

Another imperative of the pharmacist in regard to the opioid epidemic is to correct any biases or stigma that may impact their care of patients. Those who struggle from substance use disorder, addiction, or who otherwise misuse alcohol, drugs, or other substances battle stigma in every walk of life, and the medical provider’s office or clinic is no exception. Whether seeing pharmacists [22], general practitioners [23], mental health practitioners [23], general internists [24], or emergency department personnel [25], confronting a medical professional’s personal biases represents a major barrier to recovery and general care. Stigma can arise in a variety of forms, including being made to feel unworthy, incompetent, dirty, or different, or being excluded from decision-making or simply denied care [42]. Such negative treatment can further damage patients’ health through an internalization of societal stigma, experienced through repeated negative interactions with healthcare and others [43].

Stigma was evident in some student pharmacist comments. This was evident in the use of stigmatizing language, including use of the term “addict” rather than the term “person with substance use disorder,” that was recommended in the classroom session as students were educated on substance use disorders as disease states rather than moral failings. Students expressed concern that clients would misuse naloxone, using it as a flotation device so that they never learn how to swim—a dangerously fallacious idea that some may throw “Narcan parties” [44], in which people use naloxone repeatedly to avoid the potentially lethal consequences of an overdose while maintaining their same patterns of use. Although the validity of such claims has been questioned, regardless of their existence or not, such a concern and judgmental tone implies the belief that the perpetuation of drug misuse outranks in importance the value of a life saved by overdose reversal.

Many students expressed a general lack of knowledge of the drug naloxone, although many buttressed such comments with an eagerness to learn (typically coded under the Education domain and subdomain for students/curriculum). Since clinical practice typically begins later in the first year of pharmacy school, naloxone and basic harm reduction principles must be introduced before this point to ensure both the learning of student pharmacists and a high quality of care for their patients.

Student pharmacists who opined on the causes of the opioid epidemic pinned a large portion of the blame on prescribing practices. Although this is by consensus a contributing factor, no one recognized equally dire underlying issues of economic stagnation, a fraying social net, or concomitant feelings of isolation or hopelessness [45]. A seminal 2015 study by Case and Deaton attributed rising mortality rates among middle-aged non-Hispanic whites to “deaths of despair” (deaths by drug overdose, suicide, or alcohol-induced liver problems) [46]. In order to be competent practitioners of public health, student pharmacists must learn about the albeit tangled web of factors forming the opioid crisis, as well as how to mitigate the effects of social and economic inequality that lead to a higher incidence of addiction and substance use disorder in certain populations.

Thankfully, many colleges of pharmacy have embraced the call for increased substance misuse and harm reduction education. At least 94 have pledged redoubled efforts to strengthening curriculum surrounding these issues. Other effective measures include a substance use-focused curriculum over all 4 years of pharmacy school [35, 36] and intensive workshops [32]. The American Association of Colleges of Pharmacy has a growing special interests group (SIG) focused on substance use education in the pharmacy curriculum; this groups’ membership provides direction to pharmacy schools across the nation on continuous improvement on harm reduction education and cross-national collaboration and support to pharmacy educators implementing harm reduction in pharmacy curricula [47].

One strength of this study is the large sample size of student pharmacists that were surveyed. With a sample size of 167 students, it was determined by the coders that a point of saturation had been reached with no new themes emerging by the end of coding. Results of this mixed-methods study, although not generalizable to all incoming student pharmacists, shed light on curricular needs in colleges of pharmacy as educators strive to integrate harm reduction into pharmacy education in a relevant and timely manner. Although this study focused on incoming pharmacy students, the reflections shared by students also illuminate barriers to integration of harm reduction in contemporary pharmacy practice.

### Limitations

As is the case with surveys and assignments given in a classroom setting, response bias could be present despite assurances that students would not be graded on their responses. Some students in their first year of study in pharmacy school have not had the opportunity to think critically about the practice implications of harm reduction, and others have been practicing in sites that either embrace or have deeply discouraged harm reduction for a number of years. The students and faculty who participated in the administration of this project have backgrounds in public health or are well-versed in harm reduction, which could have led to coding bias. Future research will evaluate best practices for curriculum development and how pharmacy student knowledge improves from a comprehensive substance use and harm reduction curriculum throughout 4 years of postgraduate study. In the meantime, the qualitative data gathered in this study provides a useful glimpse into incoming student pharmacists’ beliefs about and knowledge of harm reduction, a first step for further work to educate competent, empathetic medical providers and create healthier communities.

## Conclusion

Countering the erroneous and sometimes stigmatizing beliefs surrounding substance use is essential to a productive curriculum within any teaching institution. In order to create such a curriculum, colleges must be informed on the baseline of knowledge with which students enter their pharmacy programs, as well as any personal biases and opinions they hold. A mixed methods approach to this task effectively identifies gaps in knowledge and informs curriculum developing, ultimately resulting in pharmacists that are better trained to combat a rising epidemic and to effectively and sensitively treat patients. The student population observed within this study demonstrated significant knowledge gaps in addiction basics, overdose risk factors, and first-aid procedures. A significant bias directed against individuals suffering from substance use disorder also surfaced in the data, even in a population of individuals striving to become healthcare professionals. In spite of this bias, the majority of students surveyed believed pharmacists can play a significant role within harm reduction measures specifically related to opioid antagonists and needle exchanges.

Developing a curriculum that can accurately teach students the fundamentals and evidence base of harm reduction strategies, and the responsibility pharmacists have to the public, including the legal regulations of the profession, is an important step to creating pharmacists that are healthcare professionals who improve the overall health of their communities.
